# The health and economic impact of vaccination with 7-valent pneumococcal vaccine (PCV7) during an annual influenza epidemic and influenza pandemic in China

**DOI:** 10.1186/s12879-015-1021-x

**Published:** 2015-07-24

**Authors:** Ronald Caldwell, Craig S. Roberts, Zhijie An, Chieh-I Chen, Bruce Wang

**Affiliations:** Department of Economics, University of Michigan, 611 Tappan Street, Ann Arbor, MI 48109 USA; Health Economics and Outcomes Research, Pfizer Inc, 500 Arcola Road, Collegeville, PA 19426 USA; National Immunization Programme, Chinese Center for Disease Control and Prevention, 27 Nanwei Road, Xicheng District, Beijing, 100050 P.R. China; Health Economics and Outcomes Research, Pfizer Investment Co. Ltd, 8/F, Citic Square, 1168 Nan Jing Road (W), Shanghai, 200041 P.R. China; Elysia Group, LLC, Xiamen Street, Alley 113, No. 17-1, Floor 2, Taipei, Taiwan

**Keywords:** Pneumococcal disease, Influenza, PCV7

## Abstract

**Background:**

China has experienced several severe outbreaks of influenza over the past century: 1918, 1957, 1968, and 2009. Influenza itself can be deadly; however, the increase in mortality during an influenza outbreak is also attributable to secondary bacterial infections, specifically pneumococcal disease. Given the history of pandemic outbreaks and the associated morbidity and mortality, we investigated the cost-effectiveness of a PCV7 vaccination program in China from the context of typical and pandemic influenza seasons.

**Methods:**

A decision-analytic model was employed to evaluate the impact of a 7-valent pneumococcal vaccine (PCV7) infant vaccination program on the incidence, mortality, and cost associated with pneumococcal disease during a typical influenza season (15 % flu incidence) and influenza pandemic (30 % flu incidence) in China. The model incorporated Chinese data where available and included both direct and indirect (herd) effects on the unvaccinated population, assuming a point in time following the initial introduction of the vaccine where the impact of the indirect effects has reached a steady state, approximately seven years following the implementation of the vaccine program. Pneumococcal disease incidence, mortality, and costs were evaluated over a one year time horizon. Healthcare costs were calculated using a payer perspective and included vaccination program costs and direct medical expenditures from pneumococcal disease.

**Results:**

The model predicted that routine PCV7 vaccination of infants in China would prevent 5,053,453 cases of pneumococcal disease and 76,714 deaths in a single year during a normal influenza season.The estimated incremental-cost-effectiveness ratios were ¥12,281 (US$1,900) per life-year saved and ¥13,737 (US$2,125) per quality-adjusted-life-year gained. During an influenza pandemic, the model estimated that routine vaccination with PCV7 would prevent 8,469,506 cases of pneumococcal disease and 707,526 deaths, and would be cost-saving.

**Conclusions:**

Routine vaccination with PCV7 in China would be a cost-effective strategy at limiting the negative impact of influenza during a typical influenza season. During an influenza pandemic, the benefit of PCV7 in preventing excess pneumococcal morbidity and mortality renders a PCV7 vaccination program cost-saving.

## Background

Over the past century, China has experienced severe outbreaks of influenza in 1918, 1957, 1968, and 2009. Influenza itself can be deadly; however the increase in mortality during an influenza outbreak is also attributable to secondary bacterial infections, specifically pneumococcal disease. During the 1918 influenza pandemic, investigators at the time concluded that the majority of deaths related to the influenza outbreak were ultimately caused by complications stemming from a secondary bacterial pneumonia infection [1] and subsequent research has provided support for this initial finding [2]. The 1918 pandemic, however, occurred in an era prior to the use of antibiotics so the proportion of influenza related deaths that were attributed to secondary bacterial infection during that pandemic may overstate the expected impact of pneumococcal infection related deaths during a modern influenza pandemic outbreak. Evidence regarding more recent pandemic outbreaks of influenza, however, suggest that mortality due to secondary bacterial infection remains a primary cause of death for individuals initially suffering from influenza, even during an era where antibiotics are in widespread use. An examination of the 1957 and 1968 pandemics in the U.S. found results similar to the study of the 1918 pandemic [2]. Additionally, a study investigating the 2009 H1N1 influenza outbreak found, by examining autopsy reports, that evidence of bacterial pneumonia was detected in approximately 55 % of influenza related mortality cases [3]. The influenza vaccine remains the primary method of protection against influenza among individuals and a possible pandemic outbreak amongst the population. However, given the associated morbidity and mortality attributed to secondary pneumococcal infections, it is important to investigate possible alternative measures that can be taken in order to help mitigate some of the negative health consequences associated with influenza.

The 7-valent pneumococcal conjugate vaccine (PCV7) was approved for use in the U.S. in 2000 and has been recommended by the U.S. Centers for Disease Control and Prevention (CDC) to be included in the routine vaccination schedule for all infants in the U.S. [4]. In the years subsequent to its inclusion into the routine vaccination schedule of the U.S., there has been a substantial impact on the incidence of pneumococcal related diseases, largely attributed to the indirect effect of herd protection on the unvaccinated child and adult populations [5, 6]. A recent report by the CDC has suggested that the majority of the reductions in invasive pneumococcal disease (IPD) following the widespread use of the PCV7 vaccine in the U.S. were due to the indirect effect on the unvaccinated population by reducing the nasopharyngeal carriage of *Streptococcus pneumonia* among immunized children [7]. As a result of the effectiveness of the PCV7 vaccine since its introduction in 2000, the World Health Organization has recommended the inclusion of PCV7 in childhood vaccination programs worldwide [8].

In this study, we examined the public health and economic impact of including PCV7 into the routine immunization program in China in the context of both a typical influenza season and a severe influenza pandemic. Although there are higher valency pneumococcal vaccines available on the market in the U.S. and elsewhere, the 7-valent pneumococcal conjugate vaccine is currently the only pneumococcal vaccine approved for use in China. As a result, we focused our study on the potential inclusion of PCV7 into the Chinese national immunization program. The PCV7 vaccine contains seven serotypes: 4, 6B, 9V, 14, 18C, 19F, and 23F, which accounts for approximately 76 % of the serotypes associated with pneumococcal disease among children less than five years of age in China [9]. Given the effectiveness of the PCV7 vaccine in reducing incidence of pneumococcal disease in both the vaccinated and unvaccinated populations due to herd protection, and the link between influenza and mortality due to secondary pneumococcal infection, it is plausible that routine vaccination of infants with PCV7, either with or without the use of the influenza vaccine, may help reduce the morbidity and mortality associated with influenza during both a typical influenza season and a potential severe influenza pandemic.

## Methods

We adapted a previously developed decision-analytic model of pneumococcal disease incidence and outcomes [10] to assess the impact of incorporating the 7-valent pneumococcal vaccine (PCV7) into the childhood vaccination schedule during both a typical influenza season and an influenza pandemic in China relative to the disease burden that would exist in the absence of a pneumococcal vaccination program. The model only included cases of pneumococcal disease and pneumococcal related deaths. Morbidity and mortality from influenza without pneumococcal co-infection were not considered. In order to simulate a single season, outcomes were assessed as a cross-sectional analysis over a one-year period which was assumed to have occurred after the vaccine indirect (herd) effects have reached a steady state, approximately seven years following the initial introduction of the vaccine. Incremental costs, life-years, and quality-adjusted life-years estimated by the model reflect the lifetime consequences of events that occur during this one-year period. All future costs and health outcomes were discounted using a 3 % annual discount rate. All cost-effectiveness results were calculated from a payer perspective and only include direct medical costs associated with treatment for pneumococcal related disease as well as the cost of the vaccine and the direct cost associated with administering the vaccine. All costs were reported using 2011 Chinese Yuan. In order to facilitate comparisons with previously published cost-effectiveness studies on the PCV7 vaccine, cost-effectiveness results were also reported in U.S. dollars using exchange rates as of July 2011 (1 USD = ¥6.465) [11].

### Model structure

The clinical starting point of the model is the choice of vaccination strategy where the model compares the case of no vaccines being administered to a case with a vaccination coverage rate of 85 % among all children less than two years of age in China over a one-year period. Individuals within the model could be vaccinated or unvaccinated, depending on the vaccination strategy and age group. All individuals are considered at risk of developing influenza, may or may not receive treatment for this illness, and may subsequently develop a secondary pneumococcal related disease. The pneumococcal disease states incorporated into the model include invasive disease cases of meningitis and bacteremia caused by *Streptococcus pneumoniae*, all-cause inpatient pneumonia, and acute otitis media. Incidence rates for pneumococcal disease states differ depending on a variety of factors: the probability of individuals within the model entering into one of the pneumococcal related disease states during the one-year period is determined by the initial choice of vaccination strategy, if the individual has developed influenza and whether they received treatment for that influenza, and the age group to which the individual belongs. Clinical outcomes within the model are assumed to be mutually exclusive. Additionally, in order to capture the full benefits of the vaccine, both the direct benefits among the vaccinated and the indirect benefits accruing to the unvaccinated population as a result of herd protection are included in the model.

### Model parameters and input data

The primary input parameters used in the model estimation, broken down by age group, are presented in Table 1 and are briefly described in the following sections.

**Table 1:** Input parameters

Parameter	Age group (Years)					
	0 - < 2	2 - 4	5 - 17	18 - 49	50 - 64	65+
Annual Incidence Rates per 100,000						
Pneumococcal meningitis	2.5	0.6	14.6	5.2	3.4	6.9
Pneumococcal bacteremia	4.8	4.7	48.8	16.7	9.2	36.0
All-cause inpatient pneumonia	4239	3830	664	164	443	3700
All-cause otitis media (per person)	0.22	0.23				
Percent of Meningitis that results in deafness	13 %	13 %	6 %	13 %	13 %	13 %
Percent of Meningitis that results in disability	7 %	7 %	5 %	7 %	7 %	7 %
Case-fatality rates						
Pneumococcal meningitis	.083	.083	.088	.472	.636	.768
Pneumococcal bacteremia	.115	.036	.024	.130	.177	.288
All-cause inpatient pneumonia	.048	.004	.008	.045	.057	.100
Direct Efficacy of Vaccine						
Pneumococcal meningitis	73.5 %	67.1 %				
Pneumococcal bacteremia	73.5 %	67.1 %				
All-cause inpatient pneumonia	16.9 %	22.9 %				
All-cause otitis media	6.4 %	5.8 %				
Indirect (herd) Effect of Vaccine						
Pneumococcal meningitis	46.8 %	40.3 %	17.5 %	38.3 %	17.4 %	33.6 %
Pneumococcal bacteremia	46.8 %	40.3 %	17.5 %	38.3 %	17.4 %	33.6 %
All-cause inpatient pneumonia	30.0 %	9.0 %	9.0 %	12.0 %	9.0 %	8.0 %
All-cause otitis media	20.1 %	19.0 %				
Cost (¥) per Case of Pneumococcal Disease						
Pneumococcal meningitis	24347	14805	24660	21825	21331	34495
Pneumococcal bacteremia	24347	14805	24660	21825	21331	34495
All-cause inpatient pneumonia	4416	2466	2867	8237	10517	14493
All-cause otitis media	148	136				

All values measured in 2011 Chinese Yuan

### Population and coverage rates

A total population of 1.34 billion was assumed for the model based on data from the Ministry of Health PRC 2009 Chinese Health Statistical Annual Report for 2010 [12]. This population was stratified into six age groups (<2, 2 to 4, 5 to 17, 18 to 49, 50 to 64, and 65 years of age and older), approximately 1.1 % of which were assumed to be children less than two years of age [12]. PCV7 is not currently included in the national immunization schedule in China and therefore the steady state level of vaccination coverage that would exist, if included, is unknown. As a result, we assumed an 85 % coverage rate that is roughly consistent with observed levels of coverage in the U.S. [13]. Each child under the age of two is assumed to have received four doses of the vaccine in the current period and children aged two to four years were assumed to have been vaccinated at some point prior to the start of the model. Consistent with previous literature, the model also assumes that there is no protection beyond the fifth year after vaccination [14, 15]. As a result, all children and adults five years of age and older were considered unvaccinated within the model.

### Disease incidence and case fatality rates

Baseline, age-specific disease incidence rates, case fatality rates, and the probabilities of developing long-term complications from pneumococcal meningitis were primarily derived from published sources. In many cases, these data were presented in age groups that did not specifically match the age groups used in this study. We combined these age groups using a weighted average based on estimated population size. Incidence and case fatality rates for pneumococcal meningitis among children less than five years of age were derived from a published meta-analysis [16, 17]. This estimate was adjusted to account for the probability that the cause of the meningitis was something other than pneumococcal infection, and to improve the estimation of disease impact by age by using data from a meta-analysis of international incidence rates and estimates of the proportion of invasive pneumococcal disease cases by age for children under five years of age in Taiwan [17–19]. Incidence and case fatality rates for IPD among individuals older than five years of age were derived from estimates of incidence from a cost-effectiveness study of lower-middle income countries [17, 20]. The probability of developing long-term complications from meningitis infection that result in deafness or disability were taken from a series of published papers [10, 14, 15, 17]. Age specific incidence and case fatality rates for inpatient pneumonia and otitis media were derived from existing data [16, 17, 20–23] with adjustments made to the age groups used in this study as described previously.

### Influenza

The cumulative influenza incidence rate over a one-year period was based on calculations from the number of specimens tested from patients with influenza like symptoms and the number of specimens found to test positive for influenza type A or B estimated from World Health Organization’s influenza virological surveillance data for China [24]. Weekly data were examined over a three year period from week forty-nine of 2010 through week forty-eight of 2013 (the last available data at the time of the calculations). These data provided an estimated one-year cumulative incidence rate of 15 %. This methodology is similar to a previously published estimate for the annual influenza incidence rate in the U.S. of 13 % using the Centers for Disease Control and Prevention’s weekly influenza surveillance data [10]. Consistent with existing literature, it was assumed that persons with influenza had a 7.7 % increased risk of developing pneumococcal disease relative to those without influenza [10, 25], and that individuals treated for influenza using antiviral medication were at 26.3 % lower risk of developing pneumococcal disease [10, 26, 27]. The proportion of those with influenza who were considered to be treated for influenza within the model was estimated to be 28.44 % based on calculations using published sources on influenza treatment in China [28, 29]. It was also assumed, consistent with previous literature, that during a severe pandemic the one-year cumulative influenza incidence rate would rise to 30 % and that there would be an increased risk of pneumococcal disease, ranging from 1.4 times to 149 times greater for bacteremic and inpatient pneumonia depending on the age group, and an increased risk of mortality stemming from pneumococcal disease relative to a typical influenza season [10].

### Vaccine efficacy and indirect effects

The vaccine efficacy and indirect effectiveness based on the herd effects estimates used in the model for all age groups are provided in Table 1. These estimates are based on a previously published study [10]. In that study, it was assumed that vaccination of children less than two years of age would result in a direct reduction in the probability of the child developing IPD based on the published efficacy of the vaccine [10, 30]. It was also assumed, consistent with published literature, that among previously vaccinated children between the ages of two and four years old that the direct benefits of the vaccine begin to wane to a level 91 % of their initial efficacy and that there is no direct protection remaining for children over the age of five years old [10, 14]. The estimated indirect (herd) effects were based on previously published data in the U.S. that measured the herd effects as a percent reduction in pneumococcal disease incidence [10, 23]. These estimates were derived by assuming that all reductions in incidence of pneumococcal infection not attributable to direct vaccine efficacy were therefore the impact of the indirect effectiveness of the vaccine.

### Cost data

All cost data incorporated into the model are Chinese specific and were based on published sources and measured in 2011 Chinese Yuan. Cost estimates for the direct, per case medical costs associated with pneumococcal infection by age group are shown in Table 1. These costs were based on a recent study that examined the cost-effectiveness of PCV7 in Shanghai [23]. In that study, the direct medical costs related to pneumococcal diseases were derived from an examination of de-identified case records from the Shanghai Board of Health Central Information Data Base and were based on confirmed inpatient cases of pneumococcal disease from hospitals in Shanghai during 2011. The total costs per case by disease type (meningitis, bacteremia, and all-cause pneumonia) and age group used in the model include hospitalization fees, physician consultation fees, diagnostic tests, operation fees, nursing fees, and medication expenses. The vaccine administration costs include the cost per dose of the vaccine, assumed to be ¥860 per dose based on published vaccine prices in Shanghai [31], and a direct administration cost assumed to be ¥10 per dose [23].

### Utility weights

Utility values used to calculate quality-adjusted-life-years (QALYs) for various disease states were taken from published sources and were incorporated into the model as a one-time reduction in the QALY for each disease occurrence. The value of the one-time reductions used in the model were 0.023 for meningitis, 0.0079 for bacteremia, 0.006 for hospitalized pneumonia, 0.004 for non-hospitalized pneumonia, and 0.005 for otitis media [32]. Utility values for deafness (0.73) and disability (0.68) resulting from meningococcal complications were also derived from published sources [33, 34] and were applied to the remaining years of life expectancy for each case.

#### Analyses

The model provides estimates for each vaccination strategy over a one-year period. Estimates were generated for the number of pneumococcal disease cases, number of deaths related to pneumococcal disease, number of estimated life years saved, the quality-adjusted-life-years (QALYs) gained, costs associated with pneumococcal disease, and costs associated with the vaccination program. Incremental cost-effectiveness ratios (ICERs) were estimated as the cost-per–life-year saved and cost-per-QALY gained for both a typical influenza season and a pandemic influenza season. Life years saved as a result of deaths avoided were calculated based on the average life expectancy remaining for each age group and were derived using life tables for China provided by World Health Organization estimates [35]. All costs and outcomes were discounted at a rate of 3 % per year.

## Results

The estimated number of pneumococcal disease cases, pneumococcal related deaths, life years, and quality-adjusted-life-years by age group predicted by the model for both the non-vaccination strategy and the strategy using PCV7 are reported in Table 2. During a normal, non-pandemic influenza season the model predicted that 114,342 cases of IPD, 1,481,050 cases of in-patient pneumonia and 76,714 deaths could be prevented as a result of routine vaccination with PCV7 among children in China. The majority of the cases prevented and the deaths averted were among the unvaccinated population due to the herd effect of the vaccine. Additionally, the model predicted that routine vaccination with PCV7 would result in 2,497,176 life years saved and 2,232,520 QALYs gained over the remaining years of life for the individuals within the model based on outcomes occurring during a single season.

**Table 2:** Estimated outcomes of disease and mortality by vaccination strategy

Parameter	Age group (Years)						
	0 - < 2	2 - 4	5 - 17	18 - 49	50 - 64	65+	Total
Non-pandemic year							
Pneumococcal meningitis (Thousands)							
No Vaccination	0.3	0.3	43.0	37.5	5.5	7.3	94.0
Vaccination	0.1	0.1	35.5	23.2	4.5	4.9	68.2
Cases Avoided	0.3	0.2	7.5	14.4	1.0	2.5	25.8
Pneumococcal bacteremia (Thousands)							
No Vaccination	0.6	2.6	144.1	118.9	14.8	38.3	319.3
Vaccination	0.1	0.7	118.9	73.4	12.2	25.5	230.8
Cases Avoided	0.5	1.9	25.2	45.5	2.6	12.9	88.6
All-cause pneumonia (Thousands)							
No Vaccination	568.6	2090.1	1959.5	1166.0	713.1	3936.9	10434.2
Vaccination	340.9	1531.3	1783.4	1026.1	648.9	3622.5	8953.1
Cases Avoided	227.6	558.8	176.2	139.8	64.2	314.4	1481.1
All-cause otitis media (Thousands)							
No Vaccination	2972.6	12476.0					15448.6
Vaccination	2285.9	9704.6					11990.5
Cases Avoided	686.7	2771.4					3458.1
Deaths							
No Vaccination	27393	8479	22921	85581	46750	410341	601465
Vaccination	16385	6156	20244	66622	42034	373311	524750
Deaths Avoided	11008	2323	2677	18959	4717	37030	76714
Life Years (Millions)							
No Vaccination	1076.3	4194.8	19298.9	31151.6	3715.1	903.1	60339.7
Vaccination	1077.2	4195.0	19299.1	31152.4	3715.2	903.4	60342.2
Life Years Saved	0.9	0.2	0.2	0.8	0.1	0.3	2.5
QALYs (Millions)							
No Vaccination	983.7	3829.8	17368.9	26696.9	2998.1	722.4	52599.8
Vaccination	984.6	3829.9	17369.1	26697.6	2998.2	722.7	52602.1
QALYs Gained	0.8	0.2	0.2	0.7	0.1	0.3	2.2
Pandemic year							
Pneumococcal meningitis (Thousands)							
No Vaccination	0.3	0.3	43.5	38.0	5.5	7.4	95.1
Vaccination	0.1	0.1	35.9	23.4	4.6	4.9	69.0
Cases Avoided	0.3	0.2	7.6	14.5	1.0	2.5	26.1
Pneumococcal bacteremia (Thousands)							
No Vaccination	3.7	38.3	6967.3	3541.4	216.6	841.1	11608.4
Vaccination	0.7	9.8	5748.2	2186.7	179.0	558.9	8683.4
Cases Avoided	2.9	28.5	1219.1	1354.7	37.6	282.2	2925.0
All-cause pneumonia (Thousands)							
No Vaccination	644.8	3096.9	3669.6	3332.9	855.9	3978.8	15578.8
Vaccination	386.7	2270.8	3371.1	2947.3	779.2	3671.2	13426.3
Cases Avoided	258.1	826.0	298.5	385.6	76.8	307.6	2152.5
All-cause otitis media (Thousands)							
No Vaccination	2986.2	12348.1					15334.3
Vaccination	2302.9	9665.5					11968.4
Cases Avoided	683.3	2682.6					3365.9
Deaths							
No Vaccination	314818	44700	779307	1170382	109165	758160	3176532
Vaccination	187101	28401	652085	869387	95870	636163	2469006
Deaths Avoided	127717	16300	127222	300995	13295	121997	707526
Life Years (Millions)							
No Vaccination	1053.2	4192.0	19249.4	31104.0	3713.7	900.1	60212.2
Vaccination	1063.5	4193.2	19257.7	31117.2	3714.0	901.1	60246.7
Life Years Saved	10.3	1.3	8.3	13.2	0.3	1.0	34.4
QALYs (Millions)							
No Vaccination	962.6	3827.2	17324.3	26656.0	2997.0	720.0	52487.1
Vaccination	972.0	3828.4	17331.8	26667.4	2997.2	720.9	52517.6
QALYs Gained	9.4	1.2	7.5	11.3	0.2	0.8	30.5

The estimated cost results are shown in Table 3. The cost savings from fewer cases of pneumococcal disease were estimated to be 12.5 billion Yuan (US $1.9 billion), and the cost of the vaccination program was 43.2 billion Yuan (US $6.7 billion) resulting in a total direct medical cost increase of 30.7 billion Yuan (US $4.7 billion) from the implementation of the vaccination program. The estimated cost-per-life-year saved was ¥12,281 (US $1,900) and the cost-per-QALY gained was ¥13,737 (US $2,125).

**Table 3:** Estimated cost (Savings) results

	No vaccinations	Vaccination	Difference
Non-pandemic year			
Disease Cost (Millions)			
Meningitis	2261.9	1648.1	(613.8)
Bacteremia	7839.5	5685.8	(2153.7)
All-Cause Pneumonia	87443.5	78171.6	(9271.9)
All-Cause otitis media	2136.7	1658.1	(478.5)
Total Disease Costs	99681.6	87163.7	(12517.9)
Vaccination Program	0	43186.4	43186.4
Total Costs	99681.6	130350.1	30668.4
Pandemic year			
Disease Cost (Millions)			
Meningitis	2287.6	1666.8	(620.8)
Bacteremia	283393.4	212734.7	(70658.7)
All-Cause Pneumonia	115122.6	102649.1	(12473.5)
All-Cause otitis media	2121.3	1655.3	(466.0)
Total Disease Costs	402924.9	318706.0	(84218.9)
Vaccination Program	0	43186.4	43186.4
Total Costs	402924.9	361892.4	(41032.5)

All values measured in 2011 Chinese Yuan

During a severe pandemic season, the model predicted that routine vaccination with PCV7 would result in 2,951,119 fewer cases of IPD, 2,152,519 fewer cases of in-patient pneumonia, and 707,526 deaths would be avoided. Approximately 46.3 % of the IPD cases prevented and 42.5 % of the deaths averted were among the 18 to 49 year old age group. The total number of life years saved and QALYs gained during a severe pandemic season were estimated to be 34.4 million and 30.5 million respectively. The direct medical cost savings were projected to be 84.2 billion Yuan (US $13.02 billion) as a result of fewer pneumococcal disease cases and 41.0 billion Yuan (US $6.34 billion) when the direct costs of the vaccination program were included. As a result, during a severe influenza pandemic, routine child vaccination with PCV7 is predicted to be more effective and less costly overall relative to the case of no vaccinations (dominant).

### Sensitivity analyses

A series of univariate sensitivity analyses were performed in order to test the robustness of the results to several key data input parameters. These analyses were performed on the level of vaccine coverage (+/− 10 %), the vaccine price (+/− 10 %), reduction of the indirect (herd) effects by 50 %, differences in base-line disease incidence rates (+/− 10 %), and differences in base-line case fatality rates (+/− 10 %) for both the typical influenza season and a severe influenza pandemic season. The results regarding the estimates for the number of deaths averted, life years saved, total direct medical costs, and cost-per-life-years saved from these analyses along with base case results are provided in Table 4.

**Table 4:** Base case and sensitivity analysis results

Parameter	Value	Deaths Averted	Life Years Saved (Millions)	Cost (Savings) (Billions)	Cost Per Life Year Saved
Non-Pandemic					
Base Case		76714	2.50	30.67	12281 (US$1899)
Vaccine Coverage					
Low	75 %	76209	2.46	24.75	10072 (US$1558)
High	95 %	77220	2.54	36.85	14525 (US$2247)
Vaccine Price					
Low (-10 %)	774	76714	2.50	26.40	10572 (US$1635)
High (+10 %)	946	76714	2.50	34.94	13991 (US$2164)
Herd Effect					
Reduced by 50 %		41195	1.47	36.17	24549 (US$3797)
Disease Incidence					
Low (-10 %)		69167	2.25	31.90	14158 (US$2190)
High (+10 %)		84524	2.75	29.40	10676 (US$1651)
Case Fatality Rate					
Low (-10 %)		69063	2.25	30.67	13640 (US$2109)
High (+10 %)		84411	2.75	30.67	11160 (US$1726)
Pandemic					
Base Case		707526	34.43	(41.03)	Dominant
Vaccine Coverage					
Low	75 %	702582	34.03	(46.88)	Dominant
High	95 %	712470	34.82	(34.93)	Dominant
Vaccine Price					
Low (-10 %)	774	707526	34.43	(45.30)	Dominant
High (+10 %)	946	707526	34.43	(36.76)	Dominant
Herd Effect					
Reduced by 50 %		384613	19.65	(0.48)	Dominant
Disease Incidence					
Low (-10 %)		641668	31.23	(33.21)	Dominant
High (+10 %)		782365	38.09	(50.06)	Dominant
Case Fatality Rate					
Low (-10 %)		637577	31.02	(41.03)	Dominant
High (+10 %)		779261	37.92	(41.03)	Dominant

All values measured in 2011 Chinese Yuan

July 2011 exchange rate, US$ = ¥6.465 [11]

Model results during a standard influenza season were most responsive to changes in the indirect (herd) effects of the vaccine on the unvaccinated population. When these effects were reduced by 50 %, the cost-per-life-year saved increased from a base case value of ¥12,281 (US$1,899) to ¥24,549 (US$3,797). The results were least sensitive to changes in case fatality rates with a cost-per-life year saved range of ¥2,480 (US$384) between the high and low values tested, followed by alterations in the vaccine price (¥3,419 (US$529)), changes in disease incidence (¥3,482 ($US539)), and changes in the level of vaccine coverage (¥4,453 (US$689)). During a severe influenza pandemic the estimates for the number of deaths averted and the life-years saved were also found to be most sensitive to changes in the indirect effects of the vaccine. In all cases, however, the cost-per-life-year saved results from including PCV7 into the routine vaccination strategy were found to remain the dominant strategy in the context of a severe influenza pandemic.

## Discussion

Several studies have examined both the clinical and economic benefits of the PCV7 vaccine in various countries in Asia [23, 36–39], however very few have specifically estimated the impact or the cost-effectiveness of including PCV7 into the routine vaccination schedule in China utilizing local economic and epidemiological data [17, 23, 40]. None of these studies, however, examine the use of PCV7 in the context of influenza. For example, in Che et al., the authors used a decision-tree model to estimate the impact of PCV7 in Chinese infants [40]. To our knowledge, there have been only two other studies that have examined the use of PCV7 in the context of both a typical influenza season and a severe pandemic [10, 41]. The existing studies used a similar theoretical framework with a focus on the U.S. population. Our study differs in that we focus on the inclusion of the PCV7 vaccine in China using, where available, local epidemiological data and Chinese specific cost data.

According to the World Health Organization’s pharmacoeconomic evaluation criteria, if the incremental cost effectiveness ratio is found to be within three times the per capita income of that region then the intervention would be considered cost-effective [42]. As of 2011, gross per capita yearly income of China was estimated to be ¥35,783 (US$5,535) [43]. In all cases, our results were found to be either cost-saving (dominant strategy) or within the recommended cost-effectiveness threshold to be considered a cost-effective intervention. The previous studies conducted on the U.S. population found the use of the pneumococcal conjugate vaccine to be a cost-saving strategy in the context of both a typical influenza season and a pandemic outbreak [10]. We also found PCV7 to be a cost-saving vaccine program during a severe influenza pandemic. However, unlike the U.S. studies, but consistent with a cost-effectiveness analysis of PCV7 in Shanghai [23], our results in a typical season were cost-effective, at ¥13,737 (US $2,125) per QALY saved. The reason for the slight differences likely has to do with differences in cost data in China, where vaccination costs are higher and treatment costs are lower than in the U.S. Note that changing the vaccination schedule would impact the costs of the program and may alter the effectiveness of the vaccines.

The study as implemented is subject to a number of potential limitations which may impact the overall results. The study was conducted from a third party payer perspective instead of using a societal perspective. As a result, we do not include the additional costs associated with pneumococcal morbidity and mortality such as the value of lost productivity from premature death, caregiver time costs, and travel costs. Given the estimated number of deaths averted and IPD cases prevented by the model, the inclusion of these costs would likely have substantially added to the potential cost savings from the use of PCV7. The study used serotype coverage data from a single study in China, but serotype coverage can vary substantially across regions and over time [9]. Were we to assume lower serotype coverage of 60 %, the projected cases averted and deaths prevented would be slightly lower. However, the overall conclusion of cost-effectiveness in a typical season and cost savings in a pandemic influenza season are still preserved. The disease cost data utilized in the model were derived from a recent study on the cost-effectiveness of PCV7 in Shanghai and are based on data from Shanghai area hospitals [23]. These data may not accurately reflect the costs associated with inpatient, pneumococcal disease in China as a whole since treatment options and price levels likely vary from region to region. We also assumed, consistent with previous literature [10, 14, 15, 17], that the direct benefits of vaccination begin to wane two years after the initial dose and that there is no protection beyond the fifth year after vaccination. As a result, all individuals within the model five years of age and older are considered unvaccinated. While consistent with previous literature, it is unlikely that the direct effectiveness of the vaccine would diminish to zero and therefore this assumption likely underestimates the potential benefits of the widespread use of PCV7 in China over time.

The analysis required extrapolation of several parameters from local and international epidemiologic data to derive necessary age-specific incidence rates. We note that the process of incorporating data derived from a variety of outside sources is subject to bias. As such, we included variations on these data in the sensitivity analyses that were performed. In all cases the results remained cost-effective under WHO thresholds of 2–3 times GDP per capita [42]. Additionally, the estimates regarding the expected increases in incidence of pneumococcal disease during a severe pandemic were derived from previously published estimates and are based on the 1918 pandemic [10]. The authors of that study, as do we, acknowledge that the 1918 pandemic was relatively more severe than subsequent pandemic outbreaks and that access to care and antibiotics has changed dramatically over the past century. As such, we regard the severe pandemic results estimated here for China as a worst case scenario and that a modern day influenza pandemic would fall somewhere within the spectrum of a typical influenza season and the severe pandemic in terms of severity of health care outcomes. In all cases, however, the estimates derived in this study were found to be cost-effective under the established World Health Organization guidelines [42].

## Conclusions

The PCV7 vaccine is effective in reducing the incidence of pneumococcal disease in both the vaccinated and unvaccinated populations due to herd protection. Given the link between influenza and mortality due to secondary pneumococcal infection, it is likely that routine vaccination with PCV7 would help reduce the morbidity and mortality associated with influenza during both a typical influenza season and potential severe influenza pandemic. Our model suggests that routine vaccination with PCV7 among infants in China would prevent more than 75,000 deaths from secondary pneumococcal infection during a typical influenza season and prevent more than 700,000 deaths and save more than 41 billion Yuan (US$6.3 billion) during a severe influenza pandemic. Given the differences in population size and cost of medical treatment, these values are consistent with previously published estimates regarding the U.S. during a severe pandemic (108,500 deaths averted and $7.34 billion in cost savings) [10]. It is likely, therefore, that the inclusion of PCV7 vaccine into the routine vaccination schedule of China would be a cost-effective intervention in the context of an influenza outbreak.

## Abbreviations

CDC: Centers for disease control and prevention
ICER: Incremental cost-effectiveness ratios
IPD: Invasive pneumococcal disease
PCV13: 13-Valent pneumococcal conjugate vaccine
PCV7: 7-Valent pneumococcal conjugate vaccine
QALY: Quality adjusted life year
USD: United states dollars
WHO: World health organization
